# GenomeBlast: a web tool for small genome comparison

**DOI:** 10.1186/1471-2105-7-S4-S18

**Published:** 2006-12-12

**Authors:** Guoqing Lu, Liying Jiang, Resa MK Helikar, Thaine W Rowley, Luwen Zhang, Xianfeng Chen, Etsuko N Moriyama

**Affiliations:** 1 Department of Biology, University of Nebraska at Omaha, Omaha, NE 68182, USA; 2 Department of Computer Science, University of Nebraska-Lincoln, Lincoln, NE 68588, USA; 3 Department of Computer Science, University of Nebraska at Omaha, Omaha, NE 68182, USA; 4 School of Biological Sciences, University of Nebraska-Lincoln, Lincoln, NE 68588, USA; 5 Nebraska Center for Virology, University of Nebraska-Lincoln, Lincoln, NE 68588, USA; 6 Virginia Bioinformatics Institute, Virginia Tech Blacksburg, VA 24061, USA; 7 Plant Science Initiative, University of Nebraska-Lincoln, Lincoln, NE 68588, USA

## Abstract

**Background:**

Comparative genomics has become an essential approach for identifying homologous gene candidates and their functions, and for studying genome evolution. There are many tools available for genome comparisons. Unfortunately, most of them are not applicable for the identification of unique genes and the inference of phylogenetic relationships in a given set of genomes.

**Results:**

GenomeBlast is a Web tool developed for comparative analysis of multiple small genomes. A new parameter called "coverage" was introduced and used along with sequence identity to evaluate global similarity between genes. With GenomeBlast, the following results can be obtained: (1) unique genes in each genome; (2) homologous gene candidates among compared genomes; (3) 2D plots of homologous gene candidates along the all pairwise genome comparisons; and (4) a table of gene presence/absence information and a genome phylogeny. We demonstrated the functions in GenomeBlast with an example of multiple herpesviral genome analysis and illustrated how GenomeBlast is useful for small genome comparison.

**Conclusion:**

We developed a Web tool for comparative analysis of small genomes, which allows the user not only to identify unique genes and homologous gene candidates among multiple genomes, but also to view their graphical distributions on genomes, and to reconstruct genome phylogeny. GenomeBlast runs on a Linux server with 4 CPUs and 4 GB memory. The online version of GenomeBlast is available to public by using a Web browser with the URL .

## Background

With the rapidly increasing availability of complete genome sequences, genome-wide sequence comparison has become an essential approach for finding homologous gene candidates, for identifying gene functions, and for studying genome evolution [1,2]. Genome comparison can be used to find genes that characterize unique features in a given organism such as specific phenotypic variation or particular pathogenicity [3]. Meanwhile, genome phylogenies based on gene content or gene order shed new light on the construction of the Tree of Life [4,5].

Currently many tools such as MUMmer and Artemis are available for comparative genomic analysis [2,6-8]. These tools can be used for pairwise genome alignment (e.g., [3,9,10]) as well as multiple genome alignment e.g., [11,12]). Unfortunately, most of them are not applicable for the identification of unique genes in a given set of genomes, since the tools were developed for homologous gene detection in most cases. Additionally, only a few tools can be used for the study of phylogeny from the genomic point of view [13].

The BLAST (Basic Local Alignment Search Tool) algorithm as well as other anchor-based algorithms are commonly used for the identification of homologous gene candidates across diverse genomes [2,14]. Although the BLAST algorithm has its pros such as fast computation and accurate results in detecting local highly-similar sequences regions, it sustains two cons when used to identify global sequence similarity: (1) genes that reside in local highly-similar regions can be erroneously identified as homologue candidates; and (2) multiple local hits that happen against the same subjective sequence need to be combined to obtain the overall aligned region between the query and subject sequences.

In order to solve these problems, we developed a Web tool, GenomeBlast. It performs multiple genome comparisons, identifies unique genes as well as shared (possibly homologous) genes among the genomes, and reconstructs the genome phylogeny. Identification of homologous gene candidates is done by detecting global sequence similarity using alignment coverage information. This paper describes its architecture, algorithms, and implementation. We demonstrate the practical use of GenomeBlast with an example using herpesviral genomes, and discuss its future improvement plan.

## Implementation

### Architecture

**Figure 1:**
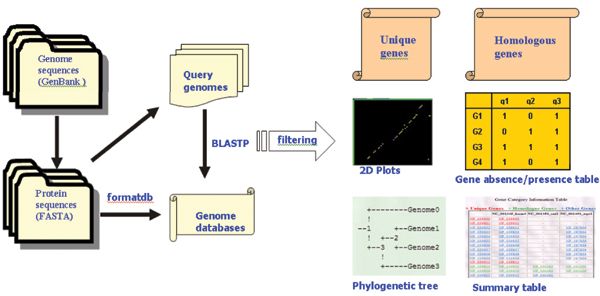
**The architecture of GenomeBlast**. GenomeBlast consists of sequence extraction, database formatting, sequence comparison, output filtering, and visual presentation of results. The inputs to GenomeBlast are genome sequences in the GenBank format, each in a single file. The outputs include three-level results: 1) putative unique genes and homologous genes; 2) 2D plots of homologous gene candidates for pairwise genome comparisons; 3) a table of gene presence/absence information, genome phylogeny, and a summary table for multiple genome comparison.

The architecture of GenomeBlast is illustrated in Figure 1. In addition to input and output modules, it consists of sequence extraction, database formatting, sequence comparison, output filtering, and visual presentation of results.

The inputs to GenomeBlast are genome sequences in the GenBank format, each in a single file. Each genome sequence record needs to include the FEATURE table with coding sequence (CDS) annotations. Such data can be downloaded from public databases such as the National Center for Biotechnology Information (NCBI)[15]. Protein sequences are extracted from translation records in the CDS annotations. The formatdb program is used to generate protein database files from the protein dataset for each genome. These protein database files can be used with the blastp program. The all-against-all blasting strategy is used for genome comparison. Each of the protein sequences from one genome is compared against protein sequences from all other genomes. The BLAST results are then filtered and presented in various outputs.

Three-level outputs generated from GenomeBlast include: (1) candidates for unique genes and homologous genes; (2) 2D plots of homologous gene candidates for pairwise genome comparisons; (3) a table of gene presence/absence information; (4) genome phylogeny; and (5) a summary table for multiple genome comparison.

### Algorithm

#### Coverage calculation

**Figure 2:**
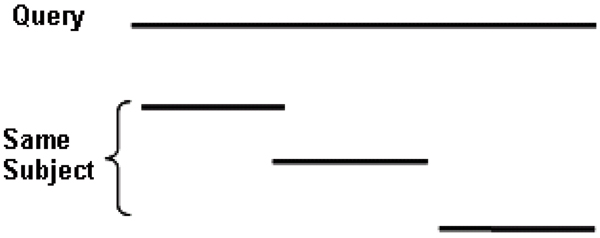
**A possible output generated by the blast program**. The blast program may find two or more highly similar regions of the same subject sequence, which need to be combined before we can evaluate global sequence similarity between the query and the subject sequence.

We used the blastp algorithm for protein sequence comparison. Since the BLAST search may result in identifying only short local similarities (short local similarities can be obtained from any conserved domains/regions even if the sequences are not derived from homologous genes) or in identifying multiple short similarities from the same CDS (Figure 2), we introduced a parameter called "coverage" to detect gene-wide sequence similarity. The percent alignment coverage (*c*) is calculated using the following equation:



where *L*_*i*_, *L*_*i*,*j*_, and *L*_*query *_represent the alignment length for the *i*^th ^hit, the overlap length between the hits *i *and *j*, and the query length, respectively; and *k *is the total number of hits to the same subject sequence for a given query sequence.

#### Identification of homologous gene candidates

In order to identify homologous gene candidates and to exclude related genes that share similarities only with limited regions, GenomeBlast can use a combination of following thresholds:

i) Coverage. The coverage is the length of aligned regions calculated as above. The default threshold is 50%.

ii) Identity. The identity is the proportion (%) of identical amino acid pairs in the aligned region. The default threshold is 30%.

iii) E-value. The E-value, expectation value, is the number of different alignments with scores equivalent to or better than the scores that are expected to occur in a database search by chance. The default threshold is 10. In the default setting, GenomeBlast uses only the coverage and identity, but not the E-value threshold.

#### Genome phylogeny reconstruction

Based on the results of multiple genome comparison, the presence and absence of each CDS is tabulated with 1s (for presence) and 0s (for absence) for each genome. Using this binary character matrix, the maximum parsimony method [16] with the branch-and-bound tree search algorithm is used to infer genome phylogeny. The branch-and-bound algorithm effectively searches the possible tree topologies and guarantees finding the most parsimonious phylogeny [17].

### Backend programs and the Web server

The blastp program in the BLAST stand-alone package  was used for protein sequence comparison. The PENNY program of the PHYLIP package implements the maximum parsimony phylogenetic method using the branch-and-bound tree search algorithm and a binary character data matrix [18]. The data processing/analysis and integration of the blastp and PENNY programs into GenomeBlast were implemented with the PERL programming language. The Web applications were developed using PHP. GenomeBlast runs on a Linux server, which has four processors (2.0 GHz each), 4 GB memory, and 400 GB disk space.

## Results

We will use thirteen herpesviral genomes described in [4] as an example, and go through GenomeBlast step by step to demonstrate its functions (Figure 1).

The first step is to set up blastp options. We did not choose the filter option to mask off low compositional complexity or mask for the lookup table. We used the default values provided in GenomeBlast (E-value: 10, word size: 3, gap existence cost: 11, gap extension cost: 1, and scoring matrix: BLOSUM62).

The next step is to upload genome sequence files. We set up the number of genomes to compare as 13 and clicked the OK button. We then uploaded the 13 herpesviral genome sequence files, which were originally downloaded from NCBI in the GenBank format. The average size of these genomes was approximately 150 kb. Formatting databases and performing all-against-all blastp comparison took 5 minutes 16 seconds on our server.

**Figure 3:**
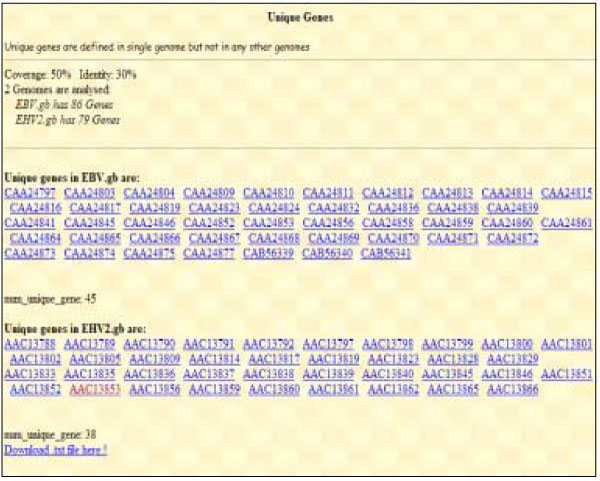
**Output window of putative unique genes**. Two alpha herpesviruses, EBV and EHV2, were selected for comparison. A total of 45 and 38 unique CDS candidates were found in EBV and EHV2, respectively.

**Figure 4:**
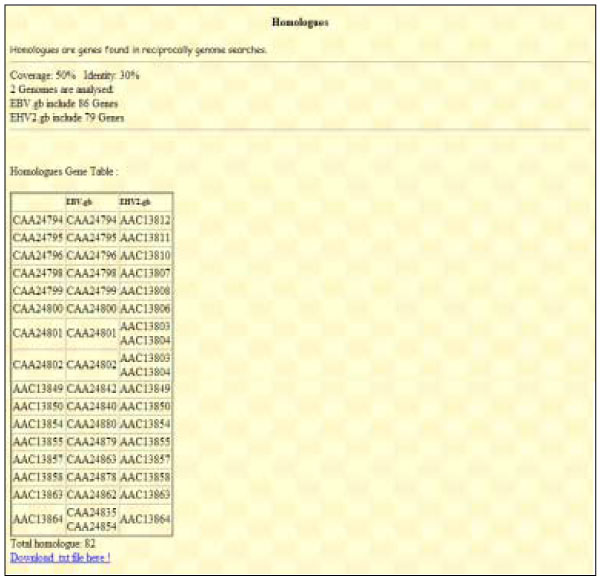
**Output window of putative homologous genes**. EBV and EHV2 were selected for comparison. 82 homologous CDS candidates were identified between them.

The third step is to set up parameters for gene comparisons. We used the default threshold values, i.e., 50% coverage and 30% identity for determining homologous CDS. The last step is to view genome comparison results at three different levels, i.e., single-genome, pairwise-genome, and multiple-genome levels. We chose two alpha viruses, EBV and EHV2, to show functions available for the single-genome level analysis. Note that any number of genome combinations can be used for unique gene or homologous gene candidate identification. A total of 45 and 38 unique gene candidates were found respectively in EBV and EHV (Figure 3), whereas 82 homologous CDS candidates were identified between these two genomes (Figure 4).

**Figure 5:**
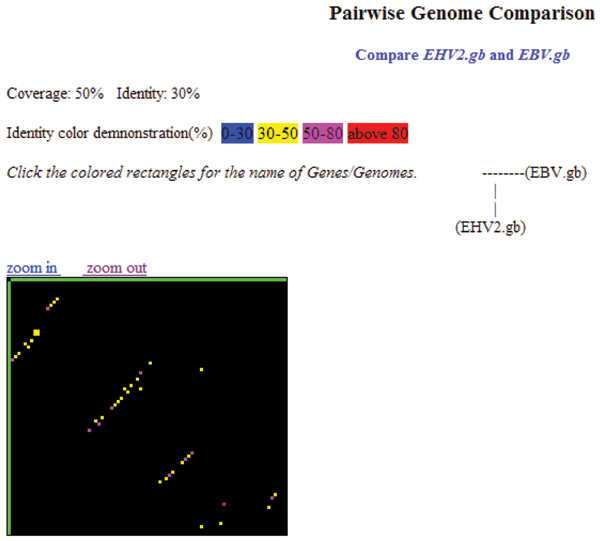
**A 2D plot of homologous gene candidates in genomes**. EBV and EHV2 were selected for comparison. The plot shows the distribution of homologous CDS on EBV and EHV2 genomes. The threshold values used for homologous CDS identification and the color scheme for identity representation are illustrated.

For the pairwise-genome comparisons, any two genomes can be chosen and a 2D plot of distribution of homologous gene candidates is generated. We clicked the hyperlink EBV.gb-EHV2.gb (alternatively, we can choose from the drop-down menu) and a 2D plot was displayed in a new window as shown in Figure 5. Interestingly, the plot suggests that genomic inversion might have occurred between these two viruses. Clicking each dot in the plot, we can see its corresponding information including the query name, subject name, and % identity. Of the 82 homologous CDS candidates, only two proteins were found to have sequence identities higher than 80% (colored in red), 20 proteins had identities between 50% and 80% (colored in pink), and the rest had identities between 30% and 50% (colored in yellow).

**Figure 6:**
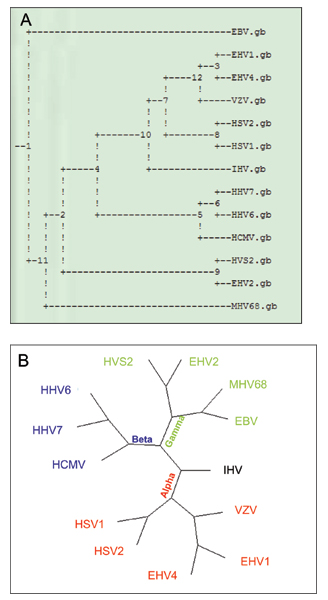
**Genome phylogeny among the herpes viruses**. The 13 herpesviral genomes described in [1, 4] were used for phylogeny inference. Panel A was generated from GenomeBlast, whereas Panel B was produced with the TreeView program using the same tree file from GenomeBlast.

At the multiple-genome level, we can obtain the binary gene presence/absence table (not shown) and the genome phylogeny as shown in Figure 6A. The phylogeny indicates that there are three virus groups, which is more clearly shown in the phylogeny redrawn with the TreeView program [19] (Figure 6B). This result showing three groups of herpesviruses is consistent with previous reports [1,4].

## Discussion

GenomeBlast has several unique features compared with other comparative genomics tools [2,3,9-12,20,21]. Instead of focusing on generating alignments, GenomeBlast identifies unique and shared, possibly homologous, CDS sets among multiple genomes and presents the information in a summary table. It generates 2D plots depicting the distribution of homologous CDS between given pairs of genomes. In order to identify possible homologous CDS, GenomeBlast uses the blastp sequence similarity search program. Combining the length of alignment coverage with % identity of the aligned region, it evaluates gene-wide similarity. This combination of coverage and identity can better identify homologous CDS candidates. GenomeBlast also provides flexibility in choosing different combinations of parameters and their threshold values. Once the blast search is done, there is no need for redoing the blast search and the user can return to the parameter-setting page to reset thresholds for identifying homologous gene candidates.

GenomeBlast reconstructs genome phylogeny based on gene content using the maximum parsimony method. In this context, GenomeBlast overlap with the Web server, SHOT [13]. SHOT also includes a gene-order phylogeny method. Whereas SHOT can be used for only a certain set of genomes, GenomeBlast offers more flexibility.

Montague and Hutchison [4] reconstructed whole-genome phylogenies for 13 herpesviral genomes based on the Clusters of Orthologous Groups (COGs) data [22]. They used several computer programs/packages before reconstructing the genome phylogenies including the Wisconsin Package (GCG) [23], BLAST programs, and PAUP (Phylogenetic Analysis Using Parsimony) [24]. We performed the same analysis using GenomeBlast alone and our genome phylogeny agreed with their result [4]. It demonstrates that GenomeBlast is a very useful application for small genome comparison. Our plan to extend functions in GenomeBlast includes automatic CDS extraction/translation, use of FASTA sequence format, DNA-level analysis using blastn, and gene-order based genome phylogeny.

GenomeBlast is suitable for small genome comparison. We do not expect it to compare large genomes, such as human and mouse genomes, because such computation with large genomes is extremely expensive, which will take several days or even weeks to complete. For larger genomes, standalone programs such as MUMmer and Artemis can be used. Or for the model organisms, some homologous gene databases such as HomoloGene [25] and Inparanoid are available for use [26-28].

## Conclusion

We have developed a Web tool for comparative analysis of small genomes. With GenomeBlast, we can identify unique genes and homologous gene candidates among multiple genomes, view their graphical distributions on genomes, and reconstruct genome phylogeny. An example with 13 herpesviral genomes demonstrated that GenomeBlast is a useful tool for genome comparison.

## Availability and requirements

• Project name: GenomeBlast project

• Project home page: 

• Operating system(s): Linux

• Programming language: PERL and PHP

• Other requirements: Any standard Web browsers (e.g., Microsoft Internet Explorer 6.0 or later)

• Any restrictions to use by non-academics: yes, contact the author GL for details

## Authors' contributions

GL conceived of the study, participated in its design and coordination, and drafted the manuscript. LJ participated in the design and implementation. RMK participated in the testing and helped to develop the Web site. TWR participated in the implementation and testing. LZ conceived of the study and helped to draft the manuscript. CZ carried out the software testing and helped to draft the manuscript. EM conceived of the study, participated in its design and coordination, and drafted the manuscript. All authors read and approved the final manuscript.
